# Anticancer agents against malaria: time to revisit?

**DOI:** 10.1016/j.pt.2009.12.002

**Published:** 2010-03

**Authors:** Alexis Nzila, John Okombo, Ruy Perez Becker, Roma Chilengi, Trudie Lang, Tim Niehues

**Affiliations:** 1 Kenya Medical Research Institute (KEMRI)/Wellcome Trust Collaborative Research Programme, PO Box 230, 80108, Kilifi, Kenya; 2 University of Oxford, Nuffield Department of Medicine, John Radcliffe Hospital, Oxford, UK; 3 Helios Klinikum Krefeld Academic Hospital, Lutherplatz 40, 47805 Krefeld, Germany

## Abstract

The emergence of artemisinin resistance could adversely impact the current strategy for malaria treatment; thus, new drugs are urgently needed. A possible approach to developing new antimalarials is to find new uses for old drugs. Some anticancer agents such as methotrexate and trimetrexate are active against malaria. However, they are commonly perceived to be toxic and thus not suitable for malaria treatment. In this opinion article, we examine how the toxicity of anticancer agents is just a matter of dose or ‘only dose makes the poison’, as coined in Paracelsus’ law. Thus, the opportunity exists to discover new antimalarials using the anticancer pharmacopoeia.

## The need for new antimalarial drugs

The malaria parasite, the cause of one of the most significant infectious diseases, is characterised by an intrinsic ability to select for resistance against drugs. To slow down the pace of resistance selection, it has been recommended that antimalarials be used in combinations of at least two drugs with different modes of action, and the current recommendation is to use artemesinin-based combinations [1]. However, recent reports indicate that resistance to artemesinin is emerging in Southeast Asia, and there are concerns that the therapeutic life span of artemisinin combinations might be compromised [2]. The choices for combination therapy could be reduced and, as a result, drug resistance would remain a limitation to sustaining effective malaria treatment.

One approach for discovering new antimalarials is to re-use drugs developed for the treatment of other diseases. The literature is replete with examples of new uses for old drugs. For example, among the few drugs that are available in the treatment of malaria, quinine is used to treat muscle cramps [3], and chloroquine (CQ) (preferably hydroxyl-chloroquine) is used for the management of rheumatoid arthritis and systemic lupus erythematosus [4]. Artemisinin is being investigated for the treatment of schistosomiasis and cancer [5,6]. However, thus far, there is no drug that has been re-used for acute malarial treatment, in spite of the richness of the human pharmacopoeia. Only drugs, such as the antibiotic doxycycline, azithromycin and erythromycin, have been used, or are currently being investigated, for prophylaxis against malaria [7,8]. Dapsone, a drug used to treat leprosy and dermatitis herpetiformis [9], was combined with chlorproguanil (Lapdap^®^) [10]; however, this combination has been phased out because of dapsone toxicity.

## Antimalarial potential of methotrexate and trimetrexate, *in vitro*

There is evidence that anticancer compounds methotrexate (MTX) and trimetrexate (TMX) are active against both pyrimethamine (PM)-sensitive and PM-resistant laboratory strains and field isolates of *Plasmodium falciparum*, including those carrying the Ileu164-Leu *dhfr* codon (dihydrofolate reductase, DHFR), with IC_50_ < 85 nM (inhibitor concentration that kills 50% of parasitaemia) [11], and IC_90/99_ values for these folate antagonist agents fall between 150 and 350 nM; thus, if such a concentration can be achieved *in vivo* with an acceptable toxicity profile, these compounds could potentially be used as antimalarials. However, anticancer agents in general, and MTX in particular, are perceived to be toxic and therefore not suitable for malaria treatment. Yet the literature is replete with examples of new uses of anticancer agents in the treatment of non-neoplastic diseases (Table 1).

## Anticancer toxicity and Paracelsus’ law

The inhibition of pathways or enzymes in tumour cells also affects normal human cells. Some anticancer agents are directed against cancer-specific targets [e.g. Imatinib mesylate (Gleevec™) targets cancer-specific tyrosine kinases] and thus can be used at doses with a relatively better toxicity profile than most drugs [12]. However, most anticancer agents are used at doses that also lead to the inhibition of growth of normal cells, in addition to blocking tumourous cells. Most specifically affected are cells that multiply actively, such as bone marrow cells, e.g. leukocytes, cells of the gastrointestinal mucosa and hair follicle cells, explaining why bone marrow suppression, mucositis and alopecia (hair loss) are among the most common side effects of anticancer compounds. However, according to Paracelsus’ law, for any drug (including anticancers), there is always a dose range at which a drug is safe.

**Figure 1:**
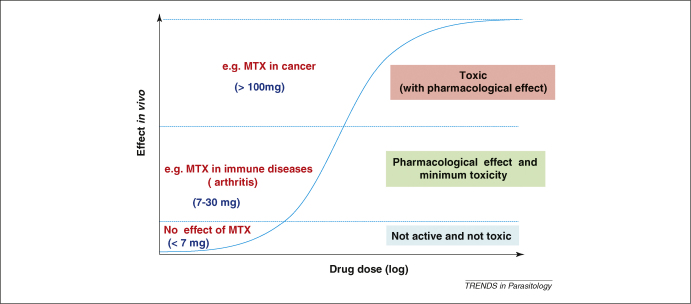
Dose–response effect of drugs in humans, as per Paracelsus’ law, using methotrexate (MTX) as an example. For any drug, there is a dose range (concentration) that is without any effect, one with a pharmacological effect but with minimal toxicity (or acceptable safety profile) and another with pharmacological and toxic effects. Most drugs used in the treatment of human diseases fall within the middle group. In the case of MTX, experience in multiple sclerosis indicates that a dose of 7.5 mg per week for up to 2 years is not associated with toxicity [21]. The use of 7–30 mg per week LD–MTX in the treatment of rheumatoid and juvenile arthritis and psoriasis is associated with an acceptable toxicity profile [19]. Higher doses (<100 mg) are associated with toxicity, as shown in the treatment of cancer [18].

Paracelsus’ law states ‘*Sola dosis facit venenum* (only dose makes the poison)’, meaning that all substances are poisons and there are none which are not. The right dose differentiates a poison from a remedy; this principle is also known as the ‘dose–response effect’ [13,14] (Figure 1). Thus, a molecule becomes a drug if the dose required to treat a complication is pharmacologically active with minimal toxicity.

The example of CQ is noteworthy. CQ is used at 10 mg kg^–1^ as a starting dose on days 1 and 2, and at 5 mg kg^–1^ on day 3 [15]. At this dose regimen, CQ has an acceptable safety profile. However, a dose of 20 mg kg^–1^ is considered toxic [15], and fatal cases have been reported from doses as low as 30 mg kg^–1^, only three times higher than the therapeutic dose [16,17]. This indicates that a slight dose increase shifts the effect of CQ from the second range (acceptable safety profile with a pharmacological effect) to the third range (life-threatening toxicity) (Figure 1). Thus, CQ has a very low safety margin, and yet it has been used widely (at the correct dose) and is considered to be one of the safest antimalarial agents available.

## Different uses of MTX in humans

MTX is another interesting example. MTX is used at high dose, up to 5000–12,000 mg per square meter (m^2^) per week (130–300 mg kg^–1^) for several weeks for the treatment of cancer, and this dose can yield serum concentrations of >1000 μM, i.e. within the range of concentrations that is associated with MTX life-threatening toxicity [18]. By contrast, a 1000-fold lower dose of MTX (LD–MTX) [0.1–0.4 mg kg^–1^ (7.5–30 mg per adult)] is used once weekly in the treatment of rheumatoid arthritis (RA), juvenile idiopathic arthritis in children (including infants <1 year old) and psoriasis [19,20].

The most common side effect of LD–MTX is mucositis (oral ulcers) and gastrointestinal (GI) tract disturbances, particularly nausea. Commonly, these adverse effects are observed after several weeks and somewhat higher doses of MTX (more than 15 mg in adults) [19]. Indeed, in the treatment of adult RA, MTX starts at a dose of 7.5 mg once weekly. After a few weeks, this dose is increased by 2.5 mg per week (the timing of the increase could vary, depending on how the patient is responding to the treatment), to reach a final dose of 25–35 mg per week. The toxicity of MTX is observed when it is used at doses >7.5 mg per week and several weeks after the first drug administration. The toxicity of MTX is a result of inhibition of the DHFR enzyme in normal cells. To prevent toxicity, a folate derivative is administered several hours after the dose of MTX. The addition of folate derivatives increases either dihydrofolate or tetrahydrofolate (DHFR substrate and product, respectively). In either case, the action of MTX against DHFR will be minimal because of the high folate content, leading to normal synthesis of pyrimidine and the restoration of cell growth. In juvenile arthritis, MTX is used at a higher dose, and there is still debate over the benefit of the addition of a folate derivative because mucositis and GI tract disturbances are rare: this drug is better tolerated by children than by adults [19], probably as a result of high cell multiplication processes in children. Overall, it is clear that the toxicity of LD–MTX result from chronic use of doses >7.5 mg per week, as has been clearly demonstrated with the use of MTX in the treatment of multiple sclerosis [21].

MTX is also being evaluated in the treatment of various disease conditions including inflammatory bowel disease [22], urticaria [23], ankylosing spondylitis [24], idiopathic hypertrophic cranial pachymeningitis [25], chronic cholestatic disorder [26], Wegener's granulomatosis [27], primary biliary cirrhosis [28], systemic lupus erythematosus [29] and inflammatory eye disease [30], haemophagocytic lymphohistiocytosis (HLH), a disease that affects younger children, including infants (<12 months of age) [31].

Worldwide, it is estimated that 0.5–1 million adults and 50,000–100,000 children receive LD–MTX weekly for the treatment of RA and juvenile idiopathic arthritis, respectively. The drug is now being used in the African population, and its safety profile is similar to that reported in the Western world [32].

## Proof of concept of MTX as an antimalarial in humans

The antimalarial potential of MTX has been established for almost 40 years. Two relatively small clinical trials, involving seven patients, have demonstrated that doses as low as 2.5 mg per day for 3–5 days were effectively treating malaria infection in humans (*Plasmodium falciparum* and/or *Plasmodium vivax*) [33,34]. However, MTX has not come into widespread use because of concerns over toxicity [35,36]. At the time of the clinical trials (in the 1970 s), no information was available on the safety of LD–MTX. Indeed, LD–MTX started to be used for the treatment of arthritis from the 1980 s; before then, MTX was used only at high doses, associated with toxicity in cancer treatment. We now have 30 years experience on the safety of LD–MTX in adults and in children, and thus this drug could now be re-evaluated as a potential antimalarial. Unlike its use in immune diseases, MTX would not be used on a chronic basis against malaria; it would be used for 3–5 days only and thus the risk of toxicity would be even lower.

The *in vivo* efficacy of LD–MTX is also supported by pharmacokinetics. Indeed, a daily dose of 5 mg in adults (0.035–0.1 mg kg^–1^) could yield serum MTX concentrations between 250 and 500 nM [37,38], concentrations which exceed the IC_90/99_ concentrations required to kill the parasite *in vitro* [11]. Taken together, the information warrants further investigation of this drug as an antimalarial.

## Potential of trimetrexate (TMX) as an antimalarial

TMX is used primarily for the treatment of solid tumours [39]. Evidence is also available that TMX has good activity against *P. falciparum*, and the addition of the folate derivative 5-methyl tetrahydrofolate (5-Me-THF) does not reduce TMX activity [40]. Thus, this form of folate could be used as an adjuvant, in combination with TMX, to increase its safety margin. 5-Me-THF would protect the host against drug toxicity and it would not negate the antimalarial activity of TMX. The same rationale has been used in the combination TMX + folinic acid (FNA) for the treatment of *Pneumocystis jiroveci* infection (an opportunistic infection commonly found in association with HIV infection). TMX is active against *P. jiroveci* and this microorganism does not salvage folate derivatives because of lack of folate receptors and transporters; thus, the addition of folate derivatives does not negate TMX activity. As a result, the combination of TMX + FNA is as active as TMX alone [41]. In cancer, a TMX dose of 100 mg per day is used for several weeks and this is associated with toxicity. In *P. jiroveci* infection, the same dose is used (100 mg per day for 28 days) but the addition of 200 mg of FNA completely reverses the toxicity of the drug, making the combination TMX + FNA well tolerated. As a result, TMX has become useful against *P. jiroveci* infection [42].

For the treatment of malaria, a much lower dose of TMX would be required. Indeed, 100 mg of TMX yields plasma concentrations of ∼5000 nM [43] and this is ∼100 times higher than the IC_50_ value (i.e. <50 nM) of TMX against *P. falciparum*. Thus, it is reasonable to propose that doses <10–20 mg of TMX would be effective to treat malaria. Such a low dose could be safe and the addition of 5-CH3-THF could even improve the therapeutic index of the drug. Although the potential exists for TMX to become an antimalarial, more work is needed to establish whether this concept could be translated *in vivo*.

## Other anticancer drugs in the treatment of malaria and other non-neoplastic diseases

We have also shown that the folate antagonist pemetrexate is active against *P. falciparum in vitro*, with activity in the nanomolar range (IC_50_ <50 nM) (A. Nzila *et al*. unpublished). Because the anticancers MTX, aminopterin and pemetrexate, all inhibitors of DHFR enzymes, are active against *P. falciparum*, other anticancer inhibitors of DHFR, such as edatrexate, pralatrexate, and piritrexim might also be active against *P. falciparum*. Several other anticancer agents have also been shown to have activity against *P. falciparum* malaria. For example, the inhibitors of the microtubulin assembly tubulozole, vinblastine, docetaxel, paclitaxel and dolastatin [44–48], the DNA crosslinking agent cisplatin [49] and the proteasome inhibitor Bortezomib [50] are effective antimalarials *in vitro*. If these drugs were active *in vivo* at low and tolerable doses, they could potentially become antimalarials. This is all the more possible in view of the fact that many anticancer drugs have already been used in the treatment of non-neoplastic diseases at low dose (Table 1).

## Concluding remarks

The burgeoning problem of antimalarial resistance highlights the need to have a strong pipeline of new drugs to treat malaria. Development of new drugs takes time and is associated with significant costs, explaining (at least partly) the paucity of available antimalarials, particularly as this is not a profitable market and thus there is little incentive for the pharmaceutical industry to develop new treatments. The re-use of existing drugs is an attractive strategy to discover new antimalarials as the development costs would be lower and the time to licensure shorter. Moreover, in the case of anticancer agents, the in-human toxicity profile would have already been well documented at much higher doses than could ever be achieved for any other product primarily developed for malaria. Many antineoplastic drugs, including MTX, were shown to be effective against the malaria parasite almost 40 years ago; however, their perceived toxicity has prevented their development as antimalarials. It is now known, and as stated by Paracelsus (more than 400 years ago), that it is ‘the dose that makes a drug’; this principle has been exploited in the use of such drugs at lower doses in several non-neoplastic diseases. It is surprising that the antimalarial potential of MTX has not been fully investigated. Exploitation of the available pharmacopoeia could be a cost-effective and efficient channel to develop much needed alternative treatments for challenging diseases such as malaria.

## Figures and Tables

**Table 1:** Selected oncologic drugs used in the treatment of non-neoplastic diseases^a^

Drug	Mechanism of action	Neoplasm (oncology)		Non-neoplasm		Refs
		Dose range^b^ and indications	Toxicity profile^c^	Dose range^b^ and indications	Toxicity profile^c^	
**MTX**		**200–2000 mg per dose**	**Grade 3**	**7.5–35 mg per week**	**Grade 0–1**	[19,20,22]
	Folate antimetabolite, inhibits DNA synthesis	ALL, breast cancer, head and neck cancer, NHL, lung cancer, osteosarcoma, Trophoblastic neoplasm	Neurological, gastrointestinal and dermatological symptoms. Pulmonary, bone marrow, renal and hepatic toxicity	Crohn's disease, rheumatoid arthritis, JIA, psoriasis, psoriatic arthritis	Gastrointestinal symptoms, transient elevation of liver enzymes, liver dysfunction
**Cyclophosphamide**	DNA alkylating agent, inhibits DNA synthesis	**250–1300 mg per dose**	**Grade 3**	**75–250 mg per dose**	**Grade 0–1**	[51]
		ALL, breast cancer, Burkitt lymphoma, HD, NHL, MM, ovarian cancer, retinoblastoma	Gastrointestinal, dermatological and catarrhal symptoms. Bone marrow, renal, hepatic, pulmonary toxicity. Acute haemorrhagic cystitis, infertility	Behcet's syndrome, idiopathic pulmonary fibrosis, ITP, JIA, lupus nephritis, NS, SLE, transplant rejection prophylaxis, multiple sclerosis, Wegener's granulomatosis	Fatigue, gastrointestinal, catarrhal and dermatological symptoms
**6-Mercapto-purine**	Purine antagonist, inhibitor of DNA and RNA synthesis	**150–350 mg per dose**	**Grade 3**	**100–150 mg per dose**	**Grade 1–2**	[52]
		ALL, AML, CML	Gastrointestinal and dermatological symptoms. Bone marrow, hepatic and renal toxicity	Crohn's disease, ulcerative colitis	Gastrointestinal symptoms, bone marrow suppression, elevation of liver enzymes
**Thalidomide**^d^	Mechanism of action is not completely understood. Selectively reduces levels of TNF	**200–1200 mg per dose**	**Grade 2–3**	**25–100 mg per dose**	**Grade 0–1**	[53]
		Kaposi's sarcoma, MM, malignant glioma, myelodysplastic syndrome, renal cell cancer	Neurological and dermatological symptoms. Bone marrow suppression, increased risk of thrombosis	Behcet's syndrome, Crohn's disease, SLE, DLE	Neurological symptoms
**Vincristine**	Vinca alkaloid: inhibitor of microtubule formation, stopping cell division	**2–3.5 mg per week**	**Grade 3**	**2 mg per month**	**Grade 0–1**	[54]
		ALL, HD, malignant glioma, neuroblastoma, NHL rhabdomyosarcoma, Wilms’ tumour	Neurological, gastrointestinal and dermatological symptoms. Bone marrow suppression, neurotoxicity	ITP,TTP	Neurological symptoms
**DFMO**^e^	Inhibitor of ornithine decarboxylase	**>10 g per dose**^e^	**Grade 4**	**0.4–0.8 g per day for a year**	**Grade 0–1**	[55,56]
Prostate cancer	Diarrhoea, abdominal pain, alopecia and ototoxicity	Chemoprotection against prostate cancer, actinic keratosis	
**>0.4 g per day for several months**	**Grade 0–1**
As a cream Hirsutism (facial hair)	
**>5 g per day for 15 days**	**Grade 1–2**
Sleeping sickness (trypanosomiasis)	Gastrointestinal symptoms
**Miltefosine**^f^	Inhibitors of phospholipid biosynthesis of cell membrane	x	x	1.5–2.5 mg per kg for 28 days Leishmaniasis	**Grade1–2**	[57]
Nausea, vomiting, diarrhoea

a Abbreviations: ALL = acute lymphocytic leukaemia, AML = acute myelogenous leukaemia, CLL = chronic lymphocytic leukaemia, CML = chronic myelogenous leukaemia, DLE = discoid lupus erythematosus, HD = Hodgkin's disease, ITP = idiopathic thrombocytopenic purpura, JIA = juvenile idiopathic arthritis, MM = multiple myeloma, NHL = non-Hodgkin's lymphoma, NS = nephritic syndrome, SLE = systemic lupus erythematosus, TTP = thrombotic thrombocytopenic purpura.

b Adult doses.

c Grade 0 = no adverse reaction, Grade 1 = mild adverse reactions, Grade 2 = moderate adverse reactions, Grade 3 = severe adverse reactions, Grade 4 = life-threatening toxicity.

d Not used in pregnancy because of its effect against foetal growth teratogenicity.

e DFMO (Difluoro-methyl-ornithine) was used as an experimental drug in cancer but was abandoned because efficacy could only be achieved with doses associated with serious life-threatening toxicity. However, the drug was revived for cancer chemoprotection, hirsutism and trypanosomiasis treatment at low dose.

f Miltefosine was initially discovered as an antineoplastic drug; however, few studies were carried out in humans to treat cancer, thus detailed information on its toxicity and dose ranges as an antineoplastic is not available.
